# Strengthening the cognition of university students to refuse e-cigarette use: A pilot randomized controlled trial of a peer-to-peer intervention

**DOI:** 10.18332/tid/208715

**Published:** 2025-09-13

**Authors:** Yu Chen, Shujun Lin, Haoxiang Lin, Zining Wang, Xinjie Zhao, Peng Ao, Yujiang Cai, Jing Xu, Xinyao Yu, Xinrui Yang, Kin-Sun Chan

**Affiliations:** 1 School of Art and Communication, Fujian Polytechnic Normal University, Fuqing, China; 2 Institute for Global Health and Development, Peking University, Beijing, China; 3 School of Journalism and Communication, Peking University, Beijing, China; 4 School of Humanities, Fujian University of Technology, Fuzhou, China; 5 School of International Studies, Peking University, Beijing, China; 6 Faculty of Humanities and Arts, Macau University of Science and Technology, Macao SAR, Macau, China; 7 Faculty of Social Sciences, University of Macau, Macao SAR, Macau, China

**Keywords:** e-cigarette prevention, peer education, Protection Motivation Theory, university students, randomized controlled trial

## Abstract

**INTRODUCTION:**

E-cigarette use among young adults represents a growing public health concern. This pilot randomized controlled trial evaluated the preliminary effects of Protection Motivation Theory (PMT)-based peer education on strengthening university students’ cognition to refuse e-cigarette use in China, where tobacco control policies remain limited.

**METHODS:**

A total of 289 participants completed baseline assessment and were randomly assigned to an intervention group (n=144) and a control group (n=145). The intervention consisted of a 1-month peer education program in which trained peer educators delivered PMT-based messages through weekly one-on-one conversations via phone or messaging platforms. Intervention participants received messages addressing all seven PMT constructs, while control participants received only messages about health risks of two PMT constructs. Primary outcomes were four PMT-based cognitive appraisals measured at baseline, 1 month, and 3 months. Linear mixed-models examined group × time interactions, and effect sizes were calculated for all comparisons.

**RESULTS:**

No statistically significant between-group differences were observed for primary outcomes. Subgroup analysis revealed significant intervention effects at 3 months among participants with family or friends who used cigarettes/e-cigarettes: lower perceived rewards (mean difference= -0.55; 95% CI: -1.07 – -0.03, p=0.04) and greater perceived efficacy (mean difference=0.34; 95% CI: 0.06–0.62, p=0.02).

**CONCLUSIONS:**

While overall effects were not statistically significant, observed effect sizes and significant subgroup findings suggest PMT-based peer education may influence cognitive precursors to e-cigarette use, particularly among students with social exposure to tobacco use. Larger trials with extended follow-up periods are warranted to confirm these preliminary findings.

## INTRODUCTION

Electronic cigarette (e-cigarette) use among young people has emerged as a significant public health concern globally^1^. In China, current e-cigarette use among young adults aged 18–24 years reached 6.5% in 2023, with higher rates among males than females^2^. In a 2021 survey of university students in in Guangzhou, overall current e-cigarette use (including dual use with cigarettes) was approximately 19.4%, again higher among males^3^. Notably, among dual users in that survey, 34.4% initiated with e-cigarettes, often during young adulthood, highlighting the critical importance of campus-based prevention efforts^3^. The diversity of device designs and appealing flavors specifically target young adults, while evidence increasingly demonstrates that e-cigarettes pose health risks including respiratory disease, cardiovascular effects, and potential for nicotine addiction^4,5^.

Preventing e-cigarette use among young adults warrants prioritization not only due to direct health effects but also because e-cigarettes may serve as a gateway to conventional cigarette use^6^. University settings provide unique opportunities for prevention, as students establish health behaviors that often persist throughout adulthood while navigating new social environments and peer influences^7^.

Peer-based interventions offer particular promise for e-cigarette prevention among university students. Young adults report greater receptivity to health messages from peers than from authority figures, perceiving peers as more understanding of their lifestyle and social pressures^8,9^. Social network influences strongly predict e-cigarette use initiation, with peer norms demonstrating stronger associations with use intentions than family norms among university students^10^. Recent evidence from peer-led e-cigarette prevention programs shows encouraging results. Chu et al.^11^ demonstrated that student opinion leaders effectively disseminated e-cigarette prevention messages in university settings, achieving significant reductions in susceptibility to use. However, most existing peer programs lack systematic theoretical frameworks and have not been evaluated in contexts with limited tobacco control infrastructure^12^.

Protection Motivation Theory (PMT) provides a comprehensive framework for understanding and influencing health behavior decisions^13^. PMT posits that behavioral intentions result from two cognitive processes: threat appraisal (perceived severity, perceived vulnerability, intrinsic rewards and extrinsic rewards) and coping appraisal (response efficacy, self-efficacy, and response costs). While PMT has demonstrated effectiveness in tobacco prevention interventions^14,15^, its application to peer-delivered e-cigarette prevention remains understudied, particularly in non-Western contexts^12^.

China presents a unique context for e-cigarette prevention research. Unlike countries with comprehensive tobacco control policies, China’s regulatory environment remains limited, with e-cigarettes widely accessible and marketed with minimal restrictions^6^. Additionally, cultural factors including collectivism and emphasis on social harmony may influence how young adults respond to peer-delivered health messages^16^. Evidence from Chinese contexts is essential for developing culturally appropriate and effective interventions.

This pilot study aimed to evaluate the preliminary effects of PMT-based peer education on strengthening university students’ cognition to refuse e-cigarette use. We hypothesized that students receiving comprehensive PMT-based messages would demonstrate stronger cognitive protection against e-cigarette use compared to those receiving only risk information. As a pilot investigation, this study sought to generate effect estimates and assess feasibility for future large-scale trials.

## METHODS

### Study design and registration

This two-arm, single-blind, randomized controlled pilot trial was conducted at Peking University. The trial was registered with the Chinese Clinical Trial Registry (ChiCTR2300068240) on 11 February 2023. The study protocol received approval from the Ethics Committee of Peking University Health Science Center (IRB00001052-23001). All participants provided written informed consent before randomization.

### Participants

Eligible participants were full-time university students aged 18–24 years who had never used cigarettes or e-cigarettes (defined as not even one puff). Exclusion criteria included diagnosed chronic conditions requiring ongoing medical treatment that might affect study participation (e.g. severe mental health conditions, conditions requiring frequent hospitalization). Recruitment occurred during 12–28 February 2023, through online advertisements, digital platforms (WeChat), and peer referrals.

### Development of intervention messages

Message development followed a systematic three-phase process grounded in PMT.

*Phase 1*

Initial message drafting based on PMT constructs. The research team developed 20–25 messages for each of seven PMT constructs, drawing from WHO reports, China’s Clinical Guidelines for Smoking Cessation, and peer-reviewed literature.

*Phase 2*

Student involvement through focus groups. Two focus groups (n=10 each) with university students assessed message comprehension, relevance, and cultural appropriateness. Students provided feedback on message clarity, believability, and potential influence on behavior.

*Phase 3*

Expert review and pilot testing. An expert panel comprising health education specialists (n=2), health communication experts (n=2), and social medicine professionals (n=2) evaluated scientific accuracy and persuasive potential. Subsequently, 20 students pilot-tested the messages, rating each on clarity (1–5 scale) and likelihood to influence behavior (1–5 scale). Table 1 presents examples of PMT-based intervention messages.

**Table 1:** Examples of PMT-based intervention messages

*PMT Construct*	*Subconstruct*	*Examples of messages*
**Perceived threat**	Perceived severity	‘Research confirms that e-cigarette use affects fetal development. Nicotine in e-cigarettes can cause birth defects, affecting heart development and lung health.’
	Perceived vulnerability	‘No matter how e-cigarettes are designed, they always contain substances harmful to humans. Young adults are particularly vulnerable to nicotine addiction.’
**Perceived rewards**	Intrinsic rewards	‘While some claim e-cigarettes help with focus, nicotine actually disrupts your natural attention systems. Try healthier alternatives like exercise or tea for genuine energy.’
	Extrinsic rewards	‘E-cigarettes don’t enhance your social life. In fact, secondhand aerosol contains harmful chemicals that others don’t want to breathe. Real friends respect healthy choices.’
**Perceived efficacy**	Response efficacy	‘Refusing e-cigarettes protects your health and sets a positive example. You can save hundreds of yuan monthly for concerts, movies, or other activities you enjoy.’
	Self-efficacy	‘Saying no to e-cigarettes is a sign of strength, not weakness. Many students successfully refuse every day – you have the same ability to protect your health.’
**Perceived costs**	Response costs	‘True friends support your healthy choices. Refusing e-cigarettes won’t damage friendships – it might even inspire others to make healthier decisions.’

### Randomization and blinding

We employed simple randomization with concealed allocation. An independent statistician prepared 20 opaque envelopes, each containing 20 folded papers (10 marked ‘1’ for intervention, 10 marked ‘2’ for control). After baseline assessment, the research coordinator drew one paper from an envelope without replacement, ensuring equal allocation while maintaining concealment. This method accommodated recruitment across multiple sites and dates. Participants remained blinded to allocation throughout the study; peer educators could not be blinded due to the nature of the intervention. This study followed the CONSORT 2010 checklist for reporting randomized trials (Supplementary file).

### Intervention

Both groups received a 1-month peer education program delivered through weekly one-on-one conversations via WeChat voice calls or messaging, based on participant preference. Each session lasted approximately 10 minutes following this structure: 2–3 minutes for check-in and rapport building; 5–6 minutes for delivery of pre-selected messages; and 2–3 minutes for participant questions and session summary.

The intervention group received messages systematically addressing all seven PMT constructs: Week 1: Perceived severity and vulnerability; Week 2: Intrinsic and extrinsic rewards (reducing perceived benefits); Week 3: Response efficacy and self-efficacy; Week 4: Response costs.

The control group received different messages about e-cigarette health risks (severity and vulnerability only) each week, ensuring equal contact time without the comprehensive PMT approach.

### Sample size

As this was a pilot feasibility study, formal *a priori* sample size calculation was not performed^17,18^. The target sample size was approximately 300 participants (150 per group), determined based on pragmatic considerations including available resources, recruitment feasibility within one academic semester, and general recommendations for pilot studies suggesting a minimum of 30–40 participants per arm for estimating preliminary effect sizes and assessing feasibility^18,19^.

### Peer educator selection and training

We recruited 20 peer educators (15 females, 5 males, aged 18–24 years) from participating universities through student health organizations. All were non-users of cigarettes and e-cigarettes, and demonstrated strong communication skills. Training consisted of: 8-hour initial workshop covering PMT theory, intervention framework, and communication skills; 4-hour practicum with standardized participant; 2-hour session on ethics and confidentiality; and weekly 30-minute supervision meetings during intervention delivery.

To prevent contamination, peer educators delivered either intervention or control messages exclusively. Each educator was assigned 10–20 participants. Fidelity monitoring included review of session documentation forms and audio recordings from 10% of randomly selected sessions.

### Outcomes and assessment

Data were collected via anonymous online questionnaires using WenJuanXing (Chinese survey platform) at baseline (before randomization), at 4 weeks (immediately post-intervention), and at 12 weeks (3 months). Participants received unique codes to link responses while maintaining anonymity.

The primary outcomes were four PMT-based cognitive appraisals measured using a validated 21-item scale^12^:

- Perceived threat: mean of perceived severity and vulnerability subscales (6 items)
- Perceived rewards: mean of intrinsic and extrinsic rewards subscales (6 items)
- Perceived efficacy: mean of response efficacy and self-efficacy subscales (6 items)
- Perceived costs: response costs subscale (3 items)

Each item used a 7-point Likert scale (1=strongly disagree to 7=strongly agree). The scale demonstrated good internal consistency in our sample: perceived severity (α=0.83), perceived vulnerability (α=0.81), intrinsic rewards (α=0.79), extrinsic rewards (α=0.77), self-efficacy (α=0.85), response efficacy (α=0.82), and response costs (α=0.78). The scale underwent cultural adaptation through expert review and cognitive interviews with 10 university students.

### Statistical analysis

The primary analysis followed intention-to-treat principles, including all randomized participants regardless of intervention completion. Prior to analysis, we assessed normality using Shapiro-Wilk tests and visual inspection of Q-Q plots. All PMT constructs showed approximately normal distributions (p>0.05), supporting parametric analyses.

Between-group comparisons at each timepoint used independent t-tests with 95% confidence intervals. Effect sizes (Cohen’s d) were calculated for all comparisons. To examine changes over time and group differences comprehensively, we employed linear mixed-models with random intercepts for participants. Fixed effects included group, time, and group × time interaction.

Pre-specified subgroup analyses examined participants with versus without family or friends who used cigarettes or e-cigarettes. We tested subgroup × treatment interactions using the Breslow-Day test. *Post hoc* power analysis using G*Power 3.1 determined achieved power for detecting small (d=0.2), medium (d=0.5), and large (d=0.8) effects. Missing data patterns were examined using Little’s MCAR test. All analyses used SPSS version 26.0 with two-sided significance at p<0.05.

## RESULTS

### Participant flow and characteristics

**Figure 1:**
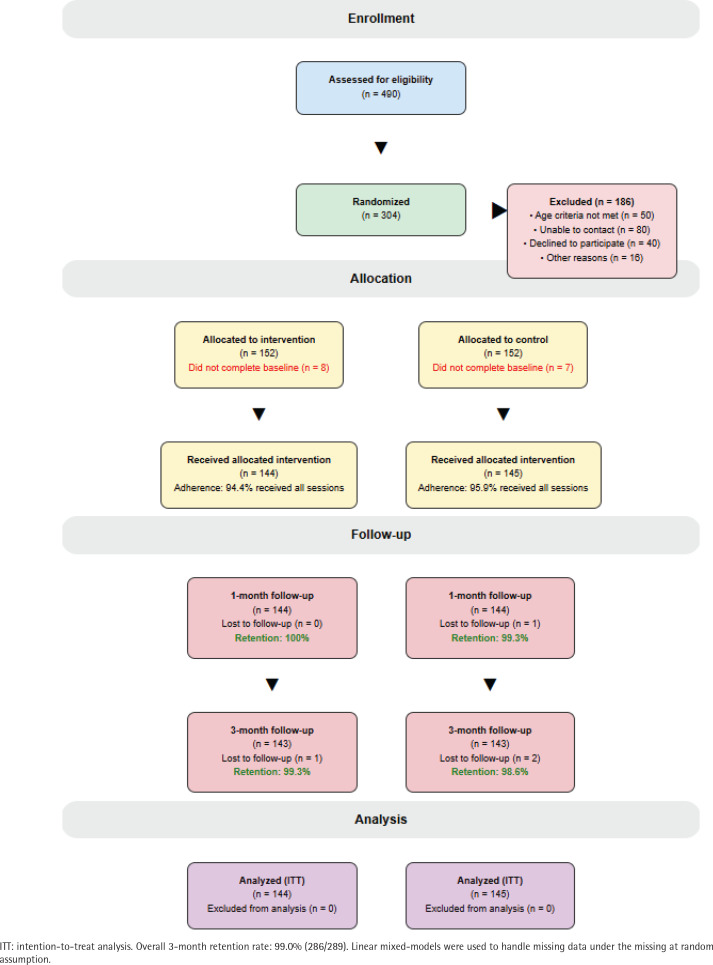
CONSORT flow diagram

Of 490 students screened, 304 met eligibility criteria and were randomized with 144 participants from 127 universities receiving the allocated intervention and 145 receiving the allocated control messages (Figure 1). Exclusions (n=186) were due to: age criteria not met (n=50), unable to be contacted (n=80), declined to participate (n=40), and other reasons (n=16). Retention was 94.1% at 1 month and 89.5% at 3 months, with similar rates between groups. The intervention was conducted in March and June 2023.

Table 2 presents sample characteristics. Groups were well-balanced, with the majority being female (75.1%), Han ethnicity (91.3%), and undergraduate students (94.5%). Mean age was 20.8 years (SD=1.6). No significant baseline differences existed between groups for demographic variables or PMT constructs (all p>0.05).

**Table 2:** Characteristics of the participants (N=289)

*Characteristics*	*Categories*	*Control group* *(N=145)* *n (%)*	*Intervention group* *(N=144)* *n (%)*	*Total* *(N=289)* *n (%)*	*χ^2^*	*p*
**Sex**	Male	35 (24.1)	37 (25.7)	72 (24.9)	0.09	0.76
	Female	110 (75.9)	107 (74.3)	217 (75.1)		
**Education level**	Undergraduate	137 (94.5)	136 (94.4)	273 (94.5)	0.00	0.99
	Graduate	8.0 (5.5)	8.0 (5.6)	16 (5.5)		
**Ethnicity**	Han	130 (89.7)	134 (93.1)	264 (91.3)	1.06	0.30
	Other	15 (10.3)	10 (6.9)	25 (8.7)		
**Monthly spending** (RMB)	<1501	43 (29.7)	30 (20.8)	73 (25.3)	5.11	0.08
	1501–2500	89 (61.4)	91 (63.2)	180 (62.3)		
	>2500	13 (9.0)	23 (16.0)	36 (12.5)		
**Parents’ cigarette use**	One/both	89 (61.4)	86 (59.7)	175 (60.6)	0.08	0.77
	None	56 (38.6)	58 (40.3)	114 (39.4)		
**Parents’ e-cigarette use**	One/both	1.0 (0.7)	6.0 (4.2)	7.0 (2.4)	3.70	0.06
	None	144 (99.3)	138 (95.8)	282 (97.6)		
**Friends’ cigarette use**	None	69 (47.6)	61 (42.4)	130 (45.0)	0.81	0.67
	Some	73 (50.3)	80 (55.6)	153 (52.9)		
	Most	3.0 (2.1)	3.0 (2.1)	6.0 (2.1)		
**Friends’ e-cigarette use**	None	92 (63.4)	90 (62.5)	182 (63.0)	0.03	0.99
	Some	51 (35.2)	52 (36.1)	103 (35.6)		
	Most	2.0 (1.4)	2.0 (1.4)	4.0 (1.4)		

RMB: 1000 Chinese Renminbi about US$140.

### Primary outcomes

**Figure 2:**
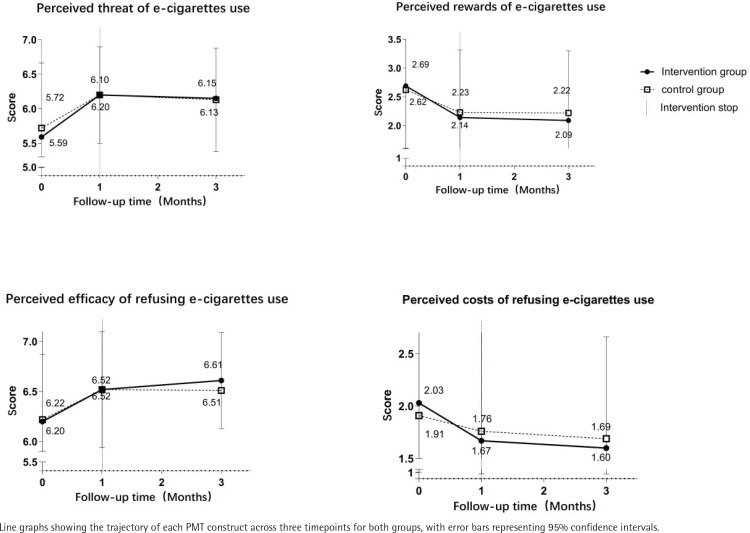
PMT construct scores over time

Figure 2 presents PMT construct scores across timepoints. Linear mixed-models revealed no significant group × time interactions for any primary outcome. At baseline, no significant differences were observed in PMT construct scores between the intervention and control groups (all p>0.05). The intervention group had a greater perceived threat of e-cigarette use and efficacy of refusing e-cigarettes and lower perceived rewards of e-cigarette use and perceived costs of refusing e-cigarettes at 3 months. However, the difference between the groups was not statistically significant.

### Subgroup analysis

**Figure 3:**
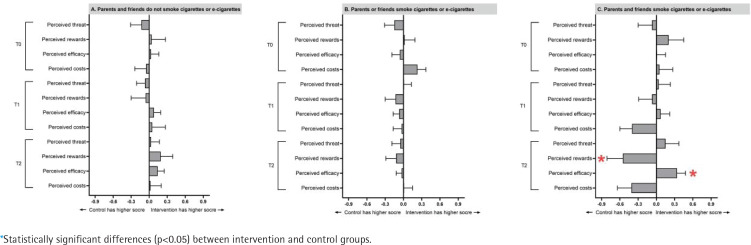
Subgroup analysis stratified by smoking status of parents and friends

To examine whether other people’s smoking/ vaping status affects cognition of e-cigarettes, we stratified the sample by parents’ and friends’ smoking and vaping status. Figure 3 and Table 3 show the mean difference in the subgroup analysis. We found that the intervention group had significantly lower perceived rewards of e-cigarette use (mean difference= -0.55; SD=0.26; p=0.04) and significantly greater perceived efficacy of refusing e-cigarettes (mean difference= 0.34; SD=0.14; p=0.02) at 3 months if they had parents and friends who use cigarettes/e-cigarettes.

**Table 3:** Subgroup analysis: participants with family/friends who use cigarettes or e-cigarettes

*Outcome at 3 months*	*Control* *Mean (SD)*	*Intervention* *Mean (SD)*	*Mean difference (95% CI)*	*p*
Perceived threat	5.48 (0.89)	5.73 (0.85)	0.25 (-0.04–0.54)	0.09
Perceived rewards	2.92 (0.94)	2.37 (0.88)	-0.55 (-1.07 – -0.03)	0.04
Perceived efficacy	5.69 (0.87)	6.03 (0.82)	0.34 (0.06–0.62)	0.02
Perceived costs	2.38 (0.88)	2.11 (0.84)	-0.27 (-0.58–0.04)	0.08

### Statistical power and effect sizes

*Post hoc* power analysis revealed that our study had 80% power to detect medium effects (d≥0.5) but only 52% power for small effects (d=0.3). The pattern of observed effect sizes, while not reaching statistical significance, showed consistency with hypothesized directions across all PMT constructs.

## DISCUSSION

This pilot randomized controlled trial provides preliminary evidence for PMT-based peer education in e-cigarette prevention among Chinese university students. While primary outcomes did not achieve statistical significance, observed effect sizes and significant subgroup findings suggest potential intervention benefits, warranting further investigation.

The lack of significant main effects requires careful interpretation. Several factors may explain these findings. First, our *post hoc* power analysis indicates insufficient power to detect small effects, which are typical for brief preventive interventions. Meta-analyses of similar programs report effect sizes of d=0.20–0.35^20^, aligning with our observed effects. Second, the control group received active health education messages about e-cigarette risks, potentially minimizing between-group differences compared to a no-treatment control. This design choice, while ethically appropriate, may have underestimated true intervention effects. Third, the 3-month follow-up may be insufficient to observe substantive cognitive changes, particularly among students without immediate e-cigarette exposure.

Significant intervention effects among participants with tobacco-using family and friends merit particular attention. This subgroup demonstrated meaningful reductions in perceived rewards and increased refusal efficacy, suggesting the intervention may be most effective for high-risk students. Social learning theory supports this finding, as students exposed to tobacco use may have stronger pre-existing positive associations requiring more intensive intervention^21^. These results align with targeted prevention approaches that allocate resources based on risk stratification.

The observed effect sizes, while modest, carry potential public health significance. Even small preventive effects can yield substantial population impact when scaled across China’s 47 million university students^15^. The intervention’s brief duration and peer delivery model enhance scalability, particularly important in resource-limited settings.

This study contributes to the limited literature on theory-based e-cigarette prevention in non-Western contexts. Despite the well-established use of theoretical frameworks (e.g. social cognitive theory, theory of planned behavior) in adolescent e-cigarette prevention programs internationally^20^, such theoretical grounding has been largely absent in most tobacco prevention activities within China^10^. This gap highlights the critical need for developing theory-driven interventions tailored to the Chinese setting. Crucially, China’s regulatory environment for tobacco control differs significantly from countries with comprehensive policies, necessitating interventions that rely less on policy support and more on individual and peer-level influences^22,23^. Our findings indicate that Protection Motivation Theory (PMT) offers a culturally adaptable framework for this purpose, particularly as its potential for peer delivery effectively capitalizes on the collectivist values prevalent in Chinese society.

### Strengths and limitations

Among the strengths of this study is the randomized controlled design with good retention that enhances internal validity. Theory-based message development with systematic student and expert input ensures content validity. The peer delivery model addresses documented preferences of young adults while building on existing social structures. Comprehensive process documentation and fidelity monitoring support reproducibility.

Several limitations warrant acknowledgment. As a pilot study, our primary aim was assessing feasibility and generating effect estimates rather than definitive efficacy testing. The relatively small sample size limited statistical power. Despite randomization, residual confounding from unmeasured variables such as personality traits, psychological characteristics, or socioeconomic factors cannot be ruled out. While we employed linear mixed-models to examine group × time interactions, the limited time points and short follow-up may have been insufficient to adequately assess cognitive change trajectories over time. Self-reported outcomes without biochemical verification introduce possible response bias, though anonymity procedures likely minimized this concern. The brief 1-month intervention may be insufficient for lasting cognitive change, suggesting future trials should extend intervention duration. Additionally, delivery via phone/messaging may reduce intensity compared to face-to-face peer interactions. The active control group receiving health education messages may have minimized between-group differences, potentially underestimating true intervention effects. Finally, peer educators could not be blinded to allocation, which may bias our results.

## CONCLUSIONS

This pilot study provides valuable preliminary evidence for PMT-based peer education in e-cigarette prevention among Chinese university students. While overall effects were not statistically significant, observed effect sizes and significant subgroup findings suggest the intervention may influence cognitive precursors to e-cigarette use, particularly among students with social exposure to tobacco use. Future trials should consider: extending follow-up periods to capture behavioral outcomes; incorporating digital delivery platforms to enhance reach while maintaining peer connection; and intensifying focus on PMT constructs showing larger effects. Implementation research examining optimal peer educator selection, training intensity, and supervision models would strengthen translation to practice. As e-cigarette use continues to rise among young adults globally, evidence-based prevention approaches adapted to local contexts remain critically needed.

## Supplementary Material



## Data Availability

The data supporting this research are available from the corresponding author on reasonable request.
